# On Abnormal Nutrition (Commonly Called Inflammation), and on the Mode in Which Its Different Products Are Developed, as Softening, Suppuration, Granulation, Reorganization of Tissue, Morbid Growths, &c. &c.

**Published:** 1843-05

**Authors:** 


*On Abnormal Nutrition (commonly called Inflammation), and on the mode in which its different Products are developed, as Softening, Suppuration, Granulation, Reorganization of Tissue, Morbid Growths, &c. &c.*—Dr. J. H. Bennett, in a communication read to the *Medico Chirurgical*

*Society of Edinburgh*, commenced by alluding to the well-known fact, that the blood circulating to every part of the living organism, carried with it the principles of nutrition. These appear to exude through the minuter vessels dissolved in the liquor sanguinis or blood plasma, which constituted a blastema or formative fluid for the formation of nucleated cells. The cells thus formed, underwent different kinds of development, some being formed into bone, others into muscle, nerve, tendon, filamentous tissue, and so on. The insensible formation and development of these cells constituted healthy nutrition.

This process might be deranged, or rendered abnormal, in various ways: 1*st*, from an increase or diminution in the whole mass of the blood; 2*dly*, from a greater or less change in the relative amount of its different chemical constituents; and, 3*dly*, from mechanical and other causes acting more especially upon any part of the frame. It was to the phenomena accompanying the latter condition that Dr. Bennett was desirous of directing the Society's attention. These were rapidly described, as they have been observed by numerous authors, and confirmed by Dr. Bennett, viz: 1*st*, Contraction of the capillaries, and diminished velocity through them of the flow of blood; 2*dly*, Dilatation of the capillaries, and diminished velocity of the blood's current; 3*dly*, Oscillation of the column of blood, and encroachment on the lymph spaces; 4*thly*, Complete stagnation of the blood, the red corpuscles crowded together in an amorphous mass, and brought into immediate contact with the vascular walls.

During the latter stage of this process, or at its termination, three circumstances might take place: 1*st*, Effusion of serum; 2*dly*, Exudation of blood plasma; and, 3*dly*, Extravasation of blood by rupture of the vessel. The object of the communication was to describe the changes which followed exudation of the liquor sanguinis.

The blood plasma on being exuded from the blood-vessels, might remain fluid for some time, and would then be necessarily reabsorbed. Vogel and Vogt refer to cases where on cutting across small cavities in the brain, the fluid they contained immediately coagulated. More frequently, however, instead of remaining fluid, the blood plasma coagulates. When this has once occurred, it undergoes changes, which vary in different cases, before it can be reabsorbed or removed from the system. The material exuded constitutes a blastema for the formation of nucleated cells, which generally, though not always, vary in character according to the nature of the tissue in which the exudation takes place.

In parenchymatous organs, the liquor sanguinis usually coagulates in the form of granules, which may be seen coating the vessels, and filling up all the space between the ultimate tissue of the organ. By this process, the organ affected is rendered perfectly dense or hepatized. After a time, or during the exudation, nucleated cells, (*exudation corpuscles*), are formed, which vary in size from 1-100th to 1-25th of a millimetre in diameter. They become filled with granules from 1-500th to 1-700th of a millimetre in diameter. The cell wall then bursts, and the granules escape. By means of this process, and the development of the exuded mass more or less into cells, it is broken up, and rendered fluid. Thus the morbid state in organs, named *softening*, is produced.

The exudation corpuscle may be distinguished by its undergoing no change on the addition of acetic acid. Ether and caustic potash entirely dissolve them; liquor ammonia renders them soft and easily broken down.

On the surface of serous membranes, the exudation generally passes into cells and very minute fibres. These cells (*plastic corpuscles*) are transparent from 1-100th to 1-75th of a millimetre in diameter, formed of a delicate wall, containing granules 1-1000th to 1-600th of a millimetre in diameter, varying in number from 3 to 12. They are not perfectly round, but somewhat irregular in form. The mode of formation of the minute fibres is unknown. Gulliver has pointed out that they are not the result of cellular development.

The plastic corpuscle may be distinguished by its wall contracting, and the edge becoming thicker on the addition of acetic acid. The shape is also rendered more irregular; it is dissolved in ether and caustic potash, and not affected by water.

In the skin, loose cellular tissue, &c., the exudation commonly passes into cells, usually from 1-100th to 1-120th of a millimetre in diameter, perfectly round, with a defined edge, containing several granules, and sometimes a round nucleus. These cells (*pus corpuscles*) swim in a fluid, roll freely on each other, are of a yellow-greenish color, and constitute the organized part of the fluid universally known as *pus*. They are not formed from the exudation corpuscle, or epithelial cells, as has been supposed, but arise primarily from the exuded blood plasma.

The pus corpuscle may be distinguished by its swelling out and becoming more transparent on the addition of water; by the cell wall being dissolved, or nearly so, in acetic acid, whilst the nucleus is rendered more distinct in the form of two or three granules, generally from 1-300th to 1-400th of a millimetre in diameter. They are dissolved in ether and concentrated alkalies.

The exudation, plastic, and pus corpuscles, although most commonly formed in the situations referred to, are not exclusively so. The pus corpuscle may sometimes be formed in parenchymatous tissues, and exudation corpuscles in cellular tissues. Sometimes they may be more or less mixed together. Thus the plastic and exudation corpuscles are commonly formed in the lung; and exudation corpuscles may frequently be found swimming among those of pus.

The exudation may also pass into *organization of tissue*, apparently by the same process as takes place in a state of health. Should it exist in small quantities, and further exudation be checked by bringing the divided parts into apposition, reorganization of tissue occurs *rapidly*, and *union by the first intention* is established. On the other hand, when this process takes place *slowly*, a state called *hypertrophy* is produced.

When loss of substance is occasioned, the exudation passes partly into organization of tissue, and partly into pus corpuscles, by means of which a *granulating surface* is produced. A fungous granulation examined under the microscope, exhibits all the stages of development presented by cells, passing into fibres, as figured by Schwann. Externally these are covered with pus corpuscles. As the former increase the latter diminish, until at length a normal tissue is reproduced, or a dense fibrous mass denominated *cicatrix*.

Lastly, the exudation may be transformed into nucleated cells of different shapes, round, oblong, caudate, stellate, more or less square, &c. &c., either mixed or unmixed with fibres, constituting the different kinds of morbid growths, as indicated by Müller.

Thus in the same manner as in a state of health, cells originating in the effused liquor sanguinis, may undergo different kinds of development, as into fibre, muscle, nerve, &c., constituting *normal* nutrition; so in a morbid state cells originate in the exuded liquor sanguinis, which are transformed into exudation, plastic, pus cells, tumours, &c., constitutihg *abnormal* nutrition.

Dr. Bennett agreed with Andral and Magendie in considering that the term inflammation was inapplicable to the explanation of the phenomena he had described. He pointed out how the cardinal symptoms of inflammation, pain, heat, redness, and swelling, were partly dependent on the exudation, and partly on the congestion which preceded it. He had even seen some cases of encephalitis, where the central parts of the brain were softened, and contained numerous exudation corpuscles, although during life no pain or heat, and after death, no redness or swelling had been observed.

Inflammation, therefore, was only a part of one great morbid action occurring in the frame, which might be denominated abnormal nutrition, and more especially that species of it dependent on increased exudation of liquor sanguinis.

Numerous authors had referred inflammation to increased nutrition or secretion. Dr. Alison more especially seemed to consider this essential to the inflammatory process, (Lib. of Med., Art. *Inflammation*). Before the doctrine of cytogenesis was established, however, nutrition of parts was invariably connected with vascularity, and pus was considered an unorganized fluid. At present we must regard pus, lymph, softening from exudation, &c., as being highly organized, and resulting from an active process of nutrition. Hitherto increased nutrition, as connected with inflammation, has been mere hypothesis; Dr. B. stated, that it was the object of his communication to *demonstrate* its correctness.—*Lond. and Eden. Month. Journ. Med. Sc.*, Dec. 1842.—*American Journ. Med. Sci.*

